# Coseismic Gravity and Displacement Signatures Induced by the 2013 Okhotsk M*w*8.3 Earthquake

**DOI:** 10.3390/s16091410

**Published:** 2016-09-01

**Authors:** Guoqing Zhang, Wenbin Shen, Changyi Xu, Yiqing Zhu

**Affiliations:** 1 School of Geodesy and Geomatics, Wuhan University, Wuhan 430079, China; zhangguo_qing_123@126.com; 2 The Second Monitoring and Application Center, China Earthquake Administration, Xi’an 710043, China; zhuyiqing@163.com; 3 State Key Laboratory of Information Engineering in Surveying, Mapping and Remote Sensing, Wuhan University, Wuhan 430079, China; 4 Institute of Earthquake Science, China Earthquake Administration, Beijing 100036, China; xuchangyi@cea-ies.ac.cn

**Keywords:** Okhotsk M*w*8.3 earthquake, GRACE, dislocation theory, coseismic gravity changes, coseismic displacements

## Abstract

In this study, Gravity Recovery and Climate Experiment (GRACE) RL05 data from January 2003 to October 2014 were used to extract the coseismic gravity changes induced by the 24 May 2013 Okhotsk M*w*8.3 deep-focus earthquake using the difference and least square fitting methods. The gravity changes obtained from GRACE data agreed well with those from dislocation theory in both magnitude and spatial pattern. Positive and negative gravity changes appeared on both sides of the epicenter. The positive signature appeared on the western side, and the peak value was approximately 0.4 microgal (1 microgal = 10^−8^ m/s^2^), whereas on the eastern side, the gravity signature was negative, and the peak value was approximately −1.1 microgal. It demonstrates that deep-focus earthquakes M*w* ≤ 8.5 are detectable by GRACE observations. Moreover, the coseismic displacements of 20 Global Positioning System (GPS) stations on the Earth’s surface were simulated using an elastic dislocation theory in a spherical earth model, and the results are consistent with the GPS results, especially the near-field results. We also estimated the gravity contributions from the coseismic vertical displacements and density changes, analyzed the proportion of these two gravity change factors (based on an elastic dislocation theory in a spherical earth model) in this deep-focus earthquake. The gravity effect from vertical displacement is four times larger than that caused by density redistribution.

## 1. Introduction

Since its launch in 2002, the Gravity Recovery and Climate Experiment (GRACE) satellites have collected more than 12 years of data, which are used for various purposes, such as determining changes in glacier mass [1,2,3], terrestrial water storage [4,5], and vertical deformation caused by seasonal hydrological loading [6,7,8]. In addition, GRACE has also detected the co- and post-seismic signatures of gravity changes resulting from huge earthquakes, including the 2004 Sumatra M*w*9.3 earthquake (thrust faults) [9,10,11,12], the 2010 Chile M*w*8.8 earthquake (thrust faults) [13,14], the 2011 Japan M*w*9.0 earthquake (thrust faults) [15,16], the 2007 Kuril M*w*8.1 earthquake (normal faults) [17], and the 2012 Indian Ocean M*w*8.7 + M*w*8.2 earthquakes (strike-slip faults) [18]. All of these earthquakes are shallow-focus earthquakes with magnitudes exceeding M*w*8.0. According to Sun et al. [19], thrust earthquakes with magnitudes greater than 7.5 and strike-slip earthquakes with magnitudes greater than 9.0 can be detected by GRACE. 

The Okhotsk M*w*8.3 deep-focus earthquake occurred on 24 May 2013, and its hypocenter depth was approximately 610 km [20,21]. This is the first earthquake greater than M*w*8 with a deep focus that occurred after the launch of GRACE. Crustal deformation has been detected by Global Positioning System (GPS) data [22]. Tanaka et al. [23] extracted the coseismic gravity steps using a time series analysis of GRACE’s monthly data spanning from February 2011 to June 2014. Besides, they analyzed the gravity contributions of the coseismic vertical deformations obtained via the half-space dislocation theory proposed by Okubo [24]. They assumed the coseismic gravity changes were dominantly invoked by the vertical deformation rather than the mass redistribution. According to their results, the coseismic gravity changes caused by the vertical deformation exceeded those caused by density changes by one order of magnitude. Their results were calculated by an elastic half-space dislocation theory proposed by Okubo [24], and they did not take into account the effects of the earth’s layered structure and the curvature of the earth. However, according to Dong et al. [25], the gravity effects of the earth’s layered structure was ~20% with source depth 20 km, and ~25% with source depth 100 km, different from the gravity effects modeled by homogeneous earth model. Hence, concerning the coseismic gravity effects caused by deep-focus earthquakes, the effects of the layered structure should be considered. 

In this study, we extracted the coseismic gravity signatures induced by the 2013 Okhotsk M*w*8.3 earthquake using different methods—GRACE’s monthly difference and time series least square fitting (LSF)—and compared the results with the corresponding theoretical predictions based on an elastic dislocation theory in a spherical earth model proposed by Sun et al. [26]. We also simulated the coseismic deformations at 20 GPS stations, analyzing the patterns of the crust deformation by combining the GPS results (GPS solutions are provided by Steblov et al. [22]). Then, we simulated the gravity contributions from grid vertical deformations and density redistribution with a grid cell size of 0.5° × 0.5° to analyze the gravity change mechanism of this earthquake. At last, we compared the results obtained by both of the two dislocation theories (based on spherical layered earth model and half-space earth model), and the results show that the elastic dislocation theory in layered earth model is necessary when calculating the deformations caused by deep-focus earthquakes. 

## 2. Coseismic Gravity Changes from GRACE

The monthly GRACE gravity solutions used to retrieve the gravity field in this study are the RL05, Level-2 products provided by the Center for Space Research (CSR, University of Texas, Austin, TX, USA) in the form of spherical harmonic (SH) coefficients with degree and order up to 60. All of the solutions contain 132 monthly data sets from January 2003 to October 2014. (Several monthly data are missing due to the problems of the GRACE satellites. Meanwhile, the data in May 2013 are removed because the earthquake occurred in that month.) RL05 products may extract stronger gravity signatures than RL04 because the former have corrections in the mean gravity field and various new tide models [27]. 

The existence of correlated errors in the GRACE data is due to the polar-orbit of the GRACE satellites and the different E–W and N–S resolutions, which cause an N–S stripes pattern in the gravity field’s spatial distribution that should be removed when extracting the gravity fields from the monthly GRACE data. In this paper, we used a decorrelation filter [28]. The basic concept is to fit the SH coefficients (order > 6) using fourth-order polynomials (P4M6) and remove the fitted results from the original SH coefficients. Moreover, we adopted the 350 km Gaussian filter [29] to reduce the effects of high-frequency noise. Because GRACE satellites are insensitive to the coefficient C_20_ (Earth’s oblateness), the C_20_ values obtained by the GRACE satellites were replaced by those obtained by satellite laser ranging (SLR) [30].

After pre-processing the GRACE data with the methods mentioned above, the time-variable gravity field could be obtained by the following formula [31]:

(1) $$\delta g(\theta ,\lambda )=\frac{GM}{r^{2}}\sum_{n=2}^{N}\left(n-1\right)\sum_{m=0}^{n}\omega \left(n\right)\left[\Delta C_{n}^{m}\cos(m\lambda )+\Delta S_{n}^{m}\sin(m\lambda )\right]{\bar{P}}_{n}^{m}\left(\cos\theta \right)$$

where *GM* is the geocentric gravitational constant, *r* is the equatorial average radius; *ω*(*n*) are the Gaussian filter coefficients, *θ* and λ are the colatitude and longitude, respectively, $\Delta C_{n}^{m}$ and $\Delta S_{n}^{m}$ are the *n*th degree and *m*th order SH coefficients, respectively, with respect to the mean gravity field from January 2003 to October 2014, and ${\bar{P}}_{n}^{m}\left(\cos\theta \right)$ is the *n*th degree and *m*th order fully normalized Legendre function.

Here, the difference method [11,32] was used to retrieve the coseismic gravity signatures. This method can weaken the effects of non-seismic seasonal factors. It is the mean gravity field from January to April 2014 minus the mean gravity field from January to April 2013 (the earthquake occurred in May 2013). The grid cell size in our calculations was 0.5° × 0.5° throughout this study.

**Figure 1:**
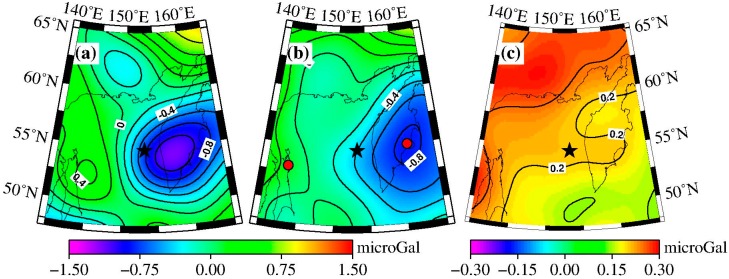
Distribution of coseismic gravity changes measured by GRACE. The P4M6 and 350 km Gaussian filter were applied. (**a**) Coseismic gravity changes retrieved by the difference method. (**b**) Coseismic gravity changes retrieved by the least square fitting (LSF). The black star represents the epicenter. The red points show the locations of the time series of the GRACE-obtained gravity changes. (**c**) Post-fitted errors of coseismic step (denoted by H in formula (2). The gravity change contour interval is 0.2 microgal in a/b, and 0.01 in (c).

The coseismic gravity changes in the spatial pattern obtained by the difference method are shown in Figure 1a. The black star represents the location of the epicenter. Figure 1a shows obvious gravity changes on both sides of the fault: the gravity signature is positive on the western side of the fault, with a peak value of 0.4 microgal, whereas the gravity signature on the eastern side is negative, with a peak value of −1.1 microgal.

We also extracted the coseismic gravity signatures using the LSF [10,23]. The time span used in this calculation is from January 2003 to October 2014. Here, we model an annual term, a semiannual term, and a 161 d S_2_ tide term as periodic signals and a constant term, a long-trend term and a coseismic jump using the following expression:

(2) $$\delta \tilde{g}=A+Bt+\sum_{i=1}^{3}C_{i}\cos\left(\omega_{i}t+\varphi_{i}\right)+\{\begin{array}{cc} 0 & t<t_{0}\\ H & t>t_{0} \end{array}$$

where $t_{0}$ is the earthquake occurrence time, and there are nine parameters, defined as follows:

(1) $C_{1}$, $\varphi_{1}$, $C_{2}$ and $\varphi_{2}$ are the amplitudes and phases of the annual and semiannual waves to model the seasonal and annual variations of hydrology and long-term oceanic circulation, respectively;
(2) $C_{3}$ and $\varphi_{3}$ are the amplitude and phase, respectively, of a 161 d sine curve used to correct the errors in the S_2_ tidal wave;
(3) *A* and *B* are the constant and linear trends of the gravity field, respectively;
(4) *H* is the coseismic jump.

The spatial pattern of coseismic gravity changes obtained by LSF is shown in Figure 1b, and the fitted errors of the coseismic gravity changes are plotted in Figure 1c. The peak values from LSF and the difference method are −0.8 to +0.3 microgal and −1.1 to +0.4 microgal, respectively. The negative peak value of the gravity changes obtained from LSF was smaller than that from difference method. Since the results from difference method were obtained by the mean gravity field from January to April 2014 minus the mean gravity field from January to April 2013, they include the post-seismic gravity changes in one year scale. Whereas the results using LSF do not contain the post-seismic effect (afterslip and viscoelastic relaxation). Besides, some differences exist in their spatial patterns. Using the difference method, the maximum negative gravity anomaly appears inland on the Kamchatka Peninsula and the minimum negative gravity anomaly is located in the northeast sea area of Sakhalin, whereas when using the LSF, the maximum negative gravity anomaly appears on the east coast of the Kamchatka Peninsula and the minimum negative gravity anomaly is located in the northeast sea area of Sakhalin along the northwest coast of Sakhalin.

**Figure 2:**
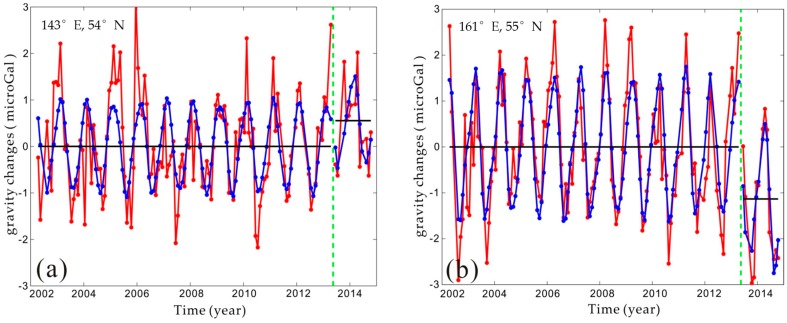
The time series at grid points (143° E, 54° N) and (161° E, 55° N) are shown by red dots in (**a**) and (**b**), respectively. The constant term and linear trend are removed. The red dots and red line represent the original gravity anomaly, and the blue dots and blue line represent the fitted gravity anomaly.

The time series of gravity changes in the peak points (i.e., A (143° E, 54° N) and B (161° E, 55° N), as shown by the red points in Figure 1b) are extracted to observe the characteristics of the gravity changes in detail, as shown in Figure 2. In Figure 2, we removed the constant and linear trend items to observe the coseismic jump, which are 0.3 and −0.8 microgal, as shown by the top and bottom plots of Figure 2, respectively. 

## 3. Modeled Coseismic Gravity Changes

**Figure 3:**
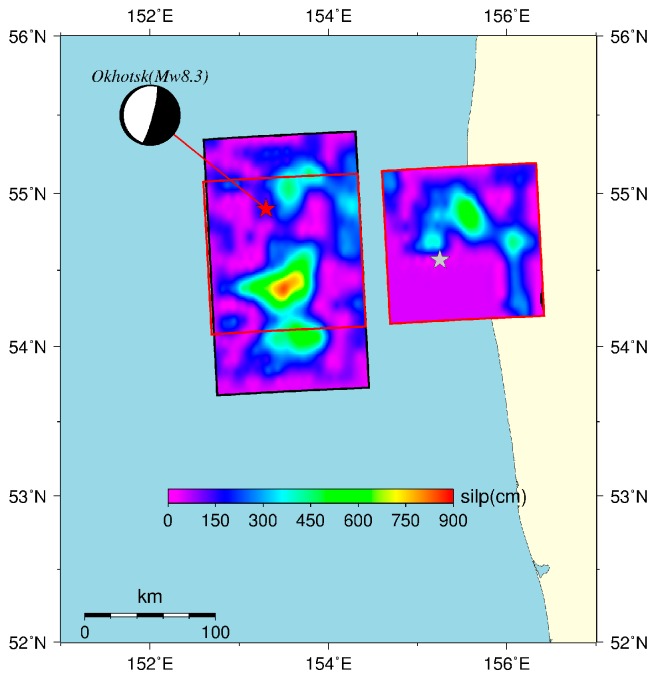
Coseismic slip model of the 2013 Okhotsk M*w*8.3 earthquake. The beach ball (top left corner) is the mechanism of the Okhotsk earthquake. The slip model includes two-stage slip model which is composed of the southward rupture (starting at the hypocenter, denoted by red star) and the northward rupture (starting at the second hypocenter, denoted by grey star). The smaller fault is located at the red rectangle in the larger fault and the maximum slip is approximately 9 m [21].

According to Sun et al. [19], a strike-slip earthquake with magnitude exceeding 9.0 and a thrust earthquake with magnitude exceeding 7.5 can be detected by GRACE satellites, and this conclusion has been partly supported by various studies [9,13,15]. However, only shallow thrust earthquakes with magnitudes M*w* > 8.5 have been detected by GRACE satellites (e.g., the 2004 Sumatra M*w*9.3 earthquake, the 2010 Chile M*w*8.8 earthquake, and the 2011 Japan M*w*9.0 earthquake). The 2013 Okhotsk M*w*8.3 earthquake is the first earthquake greater than M*w*8 with a focal depth exceeding more than 600 km after the launch of the GRACE satellites in 2002. We have extracted the gravity signature from the GRACE RL05 monthly data using the difference method and LSF (in Section 2). Considering the GRACE’s observational limitations, we modeled the coseismic gravity changes at the fixed-points near the Earth’s surface using the elastic dislocation theory proposed by Sun et al. [26] to confirm that the “coseismic gravity signatures” are real earthquake signatures rather than noises. The coseismic slip fault model was inverted by the calibrated teleseismic *P* waveforms as proposed by Wei et al. [21], and it is shown in Figure 3. The size of the fault is approximately 140 km × 50 km, containing 600 sub-faults, and the strike is 177° with dip angle 10° and maximum slip approximately 9 m.

Because the gravity changes detected by GRACE satellites are comprehensive signatures, including those induced by the redistribution of sea water, and this effect is not included in the gravity changes simulated directly by the spherical dislocation theory in a layered earth model, it therefore should be corrected. In this paper, we consider the redistribution of sea water (induced by the vertical deformation of the sea floor) as a Bouguer layer, taking into account the gravity changes, and then correct it from the original modeled result, as suggested by Zhou et al. [15]. The correction model is expressed as

(3) $$\delta g^{total}=\delta g^{solid}-2G\pi \rho_{w}hQ\left(\theta ,\lambda \right)$$

where $\delta g^{total}$ and $\delta g^{solid}$ are the seawater correction item and original results obtained by the elastic half-space dislocation theory, respectively, *G* is the gravitational constant (6.67 × 10^−11^ N·m^2^/kg), *h* is the vertical movement of Earth’s surface obtained by the dislocation theory, and Q(θ,λ) is the ocean function, which is expressed as

(4) $$Q\left(\theta ,\lambda \right)=\{\begin{array}{cc} 1 & \left(\theta ,\lambda \right)\in Ocean\\ 0 & \left(\theta ,\lambda \right)\in Land \end{array}$$

**Figure 4:**
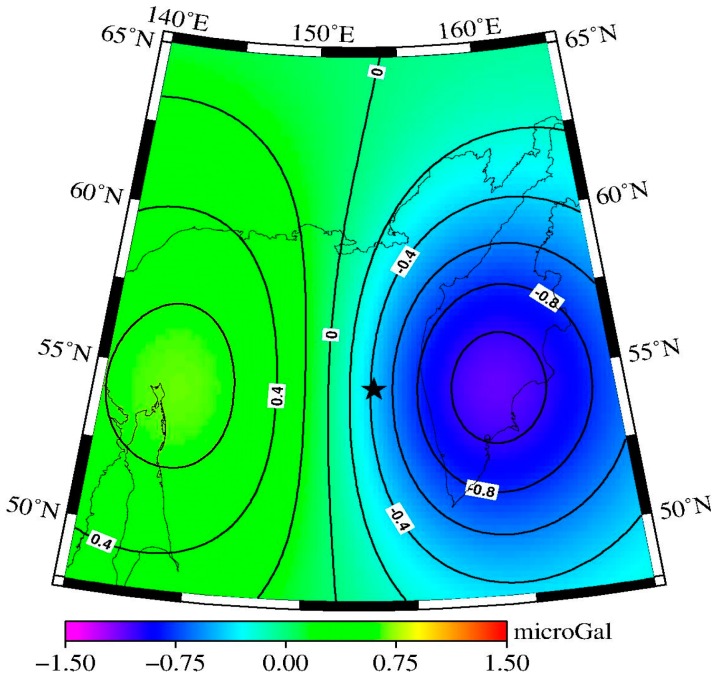
The spatial distribution of the coseismic gravity changes obtained by the elastic dislocation theory [26] in a spherical earth model after sea water correction and truncating the degree/order to 60 and applying the P4M6 decorrelated filter and 350 km Gaussian smoothing. The contour and contour-annotated intervals are 0.2 microgal and 0.4 microgal, respectively. The black star represents the location of the epicenter.

Because we applied the P4M6 decorrelated filter and 350 km Gaussian smoothing to the GRACE RL05 monthly data, and the GRACE monthly gravity field has a degree and order up to 60, we should apply the same procedure to the simulation results for comparison with the observation results, truncating the modeled field to have a degree and order up to 60 and applying P4M6 decorrelated filter and 350 km Gaussian smoothing. The post-processed coseismic gravity changes are plotted in Figure 4, where the black star represents the location of the epicenter. According to Figure 4, the gravity changes show positive–negative distribution pattern which is consistent with the observed results, and the gravity changes values range from −1.1 to +0.6 microgal.

**Figure 5:**
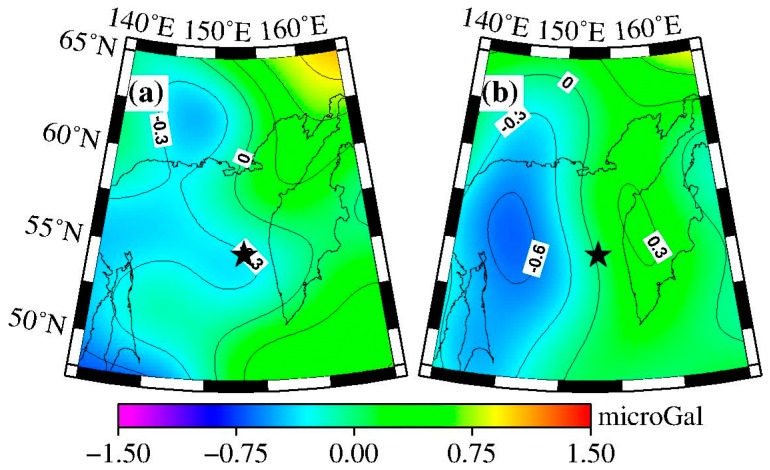
Spatial patterns of the differences between the observed results and the modeled ones: (**a**) between the difference-method results and the modeled results; and (**b**) between the LSF results and the modeled results.

In order to judge which data processing method is better when extracting the coseismic gravity change signals, comparison between the difference-method results and LSF results was made. The spatial patterns of the residual between the results using difference method and the modeled results are shown in Figure 5a (also see Figure 4 and Figure 1a), and the spatial patterns of the residual between the results using LSF and the modeled results are shown in Figure 5b (also see Figure 4 and Figure 1b). 

According to Figure 5, the gravity changes extracted by the difference method (Figure 1a) show better consistency with the model predictions (Figure 4) both in spatial pattern and magnitude. Figure 1a shows positive gravity signatures in the western side of the epicenter (north area of Sakhalin), and the maximum gravity change obtained by the difference method is 0.4 microgal, whereas the corresponding model prediction (Figure 4) is 0.6 microgal. The eastern side of the epicenter (inner Kamchatka Peninsula) shows negative gravity signatures, and the maximum gravity change obtained by the difference method is −1.1 microgal, whereas the corresponding model prediction is −1.1 microgal.

## 4. Modeled Coseismic Displacements and Gravity Contribution from Vertical Deformation

**Figure 6:**
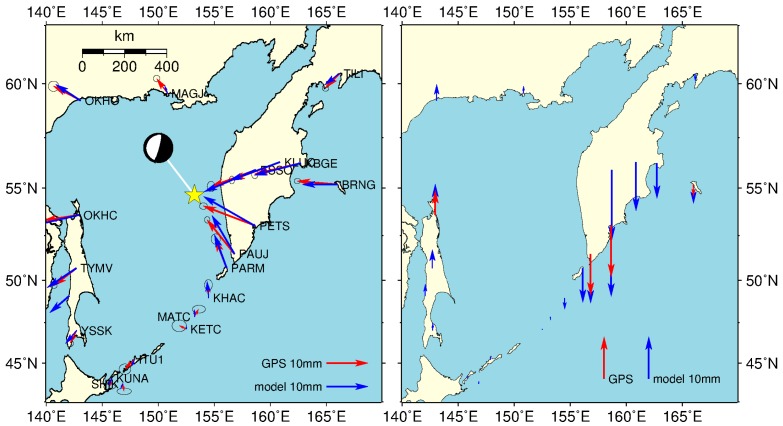
Theoretical and observed coseismic deformations. Horizontal displacement is shown in the (**left plot**), and vertical displacement is shown in the (**right plot**). The red arrows represent the Global Positioning System (GPS) results [22], and the blue arrows represent the simulated results.

In this study, we modeled the coseismic displacements at 20 GPS stations based on the dislocation theory [26] in spherical earth model, and the results are plotted in Figure 6.

**Figure 7:**
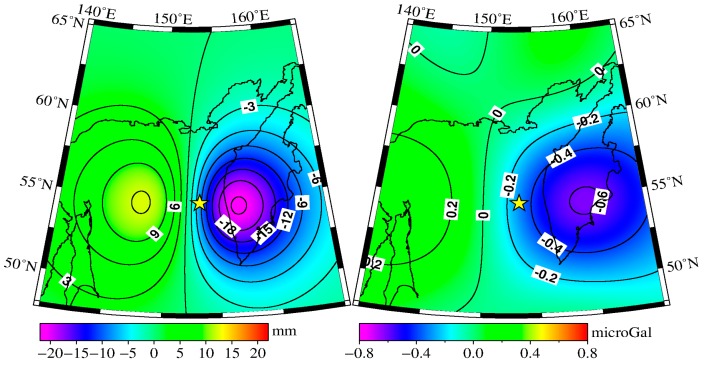
The coseismic vertical displacement (**left plot**) obtained from the dislocation theory [26], and the coseismic gravity changes (**right plot**) caused by the coseismic vertical deformation.

Our model results agree well with the GPS-observed results provided by Steblov et al. [22], as shown in Figure 6. The left plot of Figure 6 shows that on the eastern side of the epicenter (Kamchatka), both the modeled and observed results indicate that the displacements are toward the epicenter, while on the west (Kuril Islands and the north of the Okhotsk Sea), the crust motions are away from the epicenter. From the right plot of Figure 6, we can see that the crust on the west of the epicenter rises (e.g., 5.8 mm at the OKHC), while the eastern crust subsides (e.g., −12.2 mm at the PETS). We also modeled the coseismic vertical deformation with a resolution of 0.5° × 0.5° in the region 140° E–170° E, 42.5° N–62.5° N, and the spatial pattern is shown in Figure 7a. Obvious subsidence and rise occurred on both sides of the epicenter. The rise signature appears on the western side of the epicenter, and its maximum value is 12 mm, whereas on the eastern side, the crust shows subsidence signatures with maximum value of approximately 21 mm.

The coseismic gravity changes stem from two main sources [23]: (1) density redistribution near the focus caused by the fault slip; and (2) vertical displacement of the Earth’s surface and Moho. According to Tanaka et al. [23], for an earthquake with a shallow focus (~20 km), the coseismic gravity changes caused by vertical deformation have same magnitude as those from the density redistribution, and the coseismic gravity changes caused by vertical deformation should be 10 times larger than the coseismic gravity changes induced by density redistribution when the focus reaches ~600 km. The conclusions mentioned above are based on theoretical simulation by an elastic half-space dislocation proposed by Okubo [24], which did not consider the layered structure of the earth. Study of Dong et al. [25] demonstrates that the gravity effects based on the earth’s layered structure are ~20% with source depth 20 km, and ~25% with source depth 100 km, compared to the results based on the homogeneous earth model. Hence, when calculating the gravity changes caused by deep-focus earthquakes especially in this study, the earth’s layered structure should be considered. 

The coseismic gravity changes caused by vertical deformation can be expressed as

(5) $$\delta g^{vertical}=2\pi \rho Gh\left(\theta ,\lambda \right)\cdot 10^{8}$$

where $\delta g^{vertical}$ are the gravity changes caused by vertical displacements (in microgal), *ρ* is the mean density of the crust (i.e., 2700 kg/m^3^), and h(θ,λ) is the vertical displacement (in m). For comparison with the GRACE monthly differences, the gravity changes obtained by formula (5) should be expressed by a SH series truncated up to degree/order 60. The same decorrelated filter (P4M6) and 350 km Gaussian smoothing were applied to the truncated gravity changes, and the final results are shown in Figure 7b. Clear positive–negative signatures appear on both sides of the epicenter. The western side is positive, and the peak value is 0.3 microgal (located at the north of Sakhalin). Negative signatures appear to the east, and the peak value is −0.6 microgal (located in inner Kamchatka).

## 5. Gravity Contribution of Density Changes around the Source

**Figure 8:**
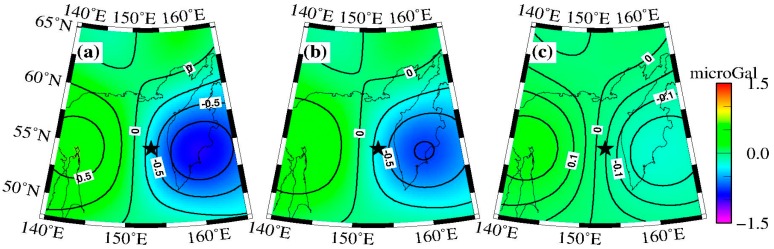
The coseismic gravity changes calculated by the elastic dislocation [24]. (**a**) Total changes; (**b**) Gravity changes caused by vertical deformation; (**c**) Gravity changes caused by density changes. The same P4M6 decorrelated filter + 350 km Gaussian smoothing was applied to GRACE data processing. The black star represents the location of the epicenter, and the contour interval is 0.25 microgal in (a) and (b), 0.1 microgal in (c).

We also obtained the coseismic gravity contributions from the density changes (modeled coseismic gravity changes minus those from vertical deformation), and the results are shown in Figure 8c (Figure 8a represents total gravity changes modeled by [26], and the Figure 8b shows the gravity changes from vertical deformation), and its peak value is approximately −0.2 microgal, which is one fourth of the peak value of the gravity changes resulting from vertical deformation. We note that this result is different from the conclusion given by [23], who stated that the coseismic gravity changes caused by vertical deformation should be 10 times larger than the coseismic gravity changes induced by density redistribution when the focus reaches ~600 km.

## 6. Conclusions and Discussion

In this paper, the coseismic gravity signatures induced by the 2013 Okhotsk M*w*8.3 earthquake are detected using different approaches: GRACE’s monthly difference method and the time series LSF. The results from LSF are smaller than those from the difference method in magnitude: the peak values of the LSF results are −0.8~+0.3 microgal, and the difference-method ones are −1.1~+0.4 microgal. We consider that this phenomenon might be due to the fact that the results based on the difference method contain post-seismic gravity changes in one year scale, while the LSF results do not include the post-seismic effects (e.g., after slip and viscoelastic effects). We also note that there is poor agreement between the spatial patterns. The negative peak value using the difference method is located in inner Kamchatka, whereas that obtained using LSF is located in the east sea of Kamchatka. To evaluate these two methods and confirm that the extracted signatures are earthquake signatures rather than noises, we modeled the coseismic gravity changes at fixed points near the Earth’s surface using the dislocation theory in a spherical earth model [26]. According to the modeled and observed results obtained by the difference method and LSF, the modeled gravity changes range from −1.1 microgal to +0.6 microgal, and the peak values are located in inner Kamchatka and north of Sakhalin. These findings are in better agreement with those of the difference method in both magnitude and spatial pattern. We note that the hydrological effect was not considered in this paper, due to the poor precision of GLDAS (Global Land Assimilation System) model and the relatively small magnitude of the coseismic gravity changes. Further, we suggest extracting the coseismic gravity change signals from GRACE monthly data by the difference method. Based on these comparisons, we conclude that GRACE satellites have successfully detected the 2013 Okhotsk M*w*8.3 deep earthquake. This is the first time that GRACE has detected the gravity changes caused by a deep-source (depth more than 600 km) earthquake, further supporting GRACE’s earthquake-monitoring capability. 

The coseismic horizontal and vertical deformations near the Earth’s surface were calculated using the elastic dislocation theory in a spherical earth model. The results agree well with GPS solutions. The stations in Kamchatka and Kuril Islands move toward the epicenter (e.g., GPS: eastward: −12.4 mm, northward: 4.7 mm; model: eastward: −12.3 mm, northward: 7.0 mm, at the PETS), and relatively large crustal subsidence occurs. In contrast, the stations in Sakhalin and along the north coast of the Okhotsk Sea moved away from the epicenter, and a small rise in the crust occurred. 

Besides, we also calculated the gravity changes caused by vertical deformation with a resolution of 0.5° × 0.5°. After application of the seawater correction and the same filter (P4M6 + 350 km Gaussian smoothing), results show that the gravity contribution of vertical deformation ranges from −0.6 to +0.3 microgal, whereas the gravity contribution of density redistribution is approximately one-fourth of the contribution of vertical deformation. Additionally, the peak value is approximately −0.2~+0.2 microgal. We modeled the gravity effects of the crust uplift/subsidence by a Bouguer layer according to Tanaka et al. [23], as well as the gravity effects of density redistribution around the fault edges by the modeled total gravity changes minus the gravity effects of the crust uplift/subsidence, and this processing method generally reflects the gravity change mechanisms according to [23]. In order to analyze the gravity change in a more precise sense, the whole spherical volume should be integrated, which was not performed in this study.

**Figure 9:**
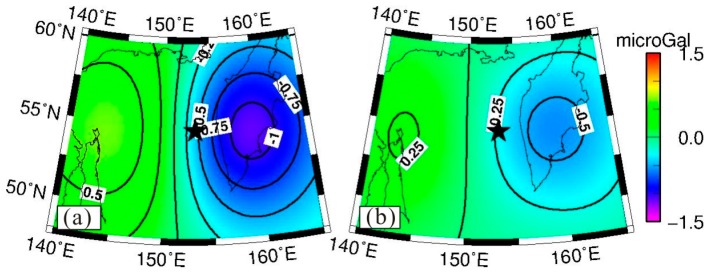
(**a**) Modeled gravity changes calculated by the dislocation theory in a spherical earth model [26]. (**b**) Modeled gravity changes calculated by the dislocation theory in a half-space earth model [24]. Seawater correction + P4M6 decorrelated filter + 350 km Gaussian smoothing has been applied to (a) and (b).

At last, according to the comparison between the observed results and the different modeled results (shown in Figure 9), as well as previous theoretical research [25], we suggest that the dislocation theory in a spherical earth model [26] should be used when calculating the coseismic deformation caused by earthquakes.
